# Modifiable Determinants of Metabolic Syndrome in Middle‐Aged Adults: A Cross‐Sectional Analysis of a Nationally Representative Health Survey

**DOI:** 10.1155/nrp/3474472

**Published:** 2026-06-09

**Authors:** Kyeongmin Jang

**Affiliations:** ^1^ Department of Nursing, College of Health Sciences, Daejin University, Pocheon-si, Gyeonggi-do, South Korea, daejin.ac.kr

**Keywords:** cardiometabolic risk, lifestyle, metabolic syndrome, middle-aged adults, nursing practice, prevention, risk factors

## Abstract

**Aims:**

To identify modifiable and nonmodifiable determinants of metabolic syndrome in adults aged 40–64 years and outline implications for nursing practice.

**Design:**

Cross‐sectional analysis of data from the 2023 Korea National Health and Nutrition Examination Survey (KNHANES), South Korea.

**Methods:**

We analyzed adults aged 40–64 years using data from the 2023 KNHANES, a nationally representative health and nutrition survey of South Korea. Metabolic syndrome was defined according to established clinical criteria. Determinants included sociodemographic, behavioral, and biological factors. Survey‐weighted analyses accounting for the complex sampling design were performed, including multivariable logistic regression to identify factors associated with metabolic syndrome. Only deidentified public data were used under existing ethical approvals.

**Results:**

Older age (≥ 50 years; OR = 2.02, 95% CI 1.60–2.55), rural residence (OR = 1.35, 95% CI 1.05–1.73), current smoking (OR = 1.48, 95% CI 1.13–1.95), and BMI ≥ 25 kg/m^2^ (OR = 8.87, 95% CI 7.21–10.90) were associated with higher odds of metabolic syndrome. In contrast, meeting physical activity recommendations (OR = 0.70, 95% CI 0.56–0.87), higher educational attainment (OR = 0.80, 95% CI 0.64–0.99), and sufficient vitamin D levels (OR = 0.79, 95% CI 0.64–0.97) were associated with lower odds.

**Conclusion:**

Determinants in middle age are largely modifiable, centering on tobacco use, physical inactivity, excess adiposity, and social context.

**Implications for the Profession and/or Patient Care:**

Nurses in primary and community settings can deliver brief counseling for smoking cessation, physical activity, weight management, and vitamin D optimization, prioritizing rural residents and adults with lower educational attainment.

**Impact:**

This study addresses the growing burden of metabolic syndrome in middle adulthood and specifies nursing‐actionable targets. The findings prioritize behavior change and weight management, with protective roles for education and vitamin D. The work can inform rapid assessment and brief interventions in primary and community care for adults aged 40–64 years, especially where rural residence and educational disadvantage are prevalent.

**Study Design and Reporting Guideline:**

This research is a cross‐sectional analysis. The STROBE checklist for cross‐sectional studies is completed and submitted.

**Statistical Compliance Statement:**

(a) Statistical methods adhered to JAN Statistical Guidelines and best practices for complex survey data. (b) The analysis accounted for the complex, multistage, stratified cluster design of KNHANES with appropriate weighting and Taylor‐series linearization for variance estimation. (c) Model assumptions were examined; effect estimates are presented with 95% confidence intervals, and confounder adjustment is described in the Methods section. (d) The author (Kyeongmin Jang, RN, PhD) served as the author‐statistician and accepts responsibility for the statistical methods and interpretation. (e) The author confirms that data coding, preprocessing, and analysis steps are documented and reproducible.

## 1. Introduction

Metabolic syndrome (MetS) is a clustering of interrelated abnormalities—central adiposity, elevated triglycerides, reduced high‐density lipoprotein cholesterol (HDL), elevated blood pressure, and impaired fasting glucose—that elevates risks for cardiovascular disease, Type 2 diabetes, chronic kidney disease, and premature mortality [1]. Diagnosis is typically made when three or more components co‐occur, reflecting underlying visceral adiposity, insulin resistance, and chronic low‐grade inflammation [2]. Globally, the burden of MetS has risen in step with population aging, urbanization, energy‐dense diets, and declining physical activity [3].

Midlife is a critical window for prevention. Metabolic risk commonly emerges during the fifth and sixth decades, yet remains amenable to behavior change before vascular and glycemic damage becomes entrenched [4]. Targeting determinants in adults aged 40–64 years can therefore yield high clinical and policy returns, particularly where primary and community care nurses are positioned to screen, counsel, and refer.

Determinants span nonmodifiable, social, behavioral, and biological domains. Age is a consistent predictor; sex patterns often show higher burden among men in midlife with convergence or reversal after menopause [5]. Education and income are frequently protective, plausibly through health literacy, access to preventive services, and neighborhood resources [6]. Place of residence also matters: Rural settings can entail longer travel to care, fewer built‐environment supports for activity, and limited prevention programs [7]. Among behaviors, cigarette smoking is linked to insulin resistance, dyslipidemia, and central fat deposition via oxidative and inflammatory pathways [8]. In contrast, meeting recommended physical activity levels improves insulin sensitivity, blood pressure, and lipid profiles while reducing visceral adiposity [9]. Sleep and diet likely contribute, though the findings vary across measures [10]. Biological indicators such as body mass index (BMI) and serum vitamin D may provide useful information for assessing cardiometabolic risk in middle‐aged adults. BMI is a well‐established marker of metabolic risk, and vitamin D status has also been linked to cardiometabolic outcomes [11–13].

Despite extensive literature, important gaps persist for midlife adults in recent, nationally representative datasets. Many studies combine broad adult age ranges, rely on convenience samples, or do not account for complex survey design, limiting generalizability and precision [14]. Moreover, modifiable behaviors are not always analyzed alongside structural factors, making it difficult to specify nursing‐actionable priorities.

This study addresses these gaps using contemporary national health examination data from the 2023 Korea National Health and Nutrition Examination Survey (KNHANES), South Korea. The aim of this study was to estimate the prevalence of MetS and identify its independent determinants among adults aged 40–64 years across sociodemographic, behavioral, and biological domains. By applying the survey’s complex design, we sought to generate population‐relevant evidence to inform feasible nurse‐led actions, including routine waist‐circumference and blood‐pressure assessment, brief counseling for smoking cessation and physical activity, weight‐management support, and consideration of vitamin D status, with particular attention to rural residents and adults with lower educational attainment, using national data from the 2023 KNHANES, South Korea. The aim of this study was to identify the prevalence and independent determinants of MetS among adults aged 40–64 years.

## 2. Methods

### 2.1. Study Design and Data Source

This study employed a secondary analysis of nationally representative cross‐sectional data from the KNHANES, conducted by the Korea Disease Control and Prevention Agency (KDCA). The 2023 KNHANES data, officially released in December 2024, were used for this analysis. KNHANES is a nationwide surveillance system that collects data through standardized health interviews, physical examinations, and nutrition surveys conducted by trained personnel. The survey uses a complex, multistage, stratified cluster sampling design to ensure representativeness of the noninstitutionalized Korean population [15].

### 2.2. Participants

A total of 6929 individuals participated in the 2023 KNHANES. Among them, 2781 participants were aged 40–64 years. After excluding those with missing data on key variables, 2580 participants were included in the final analysis. All analyses were conducted using anonymized public‐use data.

### 2.3. Independent Variables

The independent variables in this study encompassed sociodemographic, behavioral, and biological characteristics. Sociodemographic variables included age, sex, education level (≥ college graduate vs. below college), income level (upper 50% vs. lower 50%), and residential area (urban vs. rural). Behavioral variables included current smoking (yes/no), alcohol consumption in the past year (yes/no), physical activity level, and sleep duration (≥ 7 vs. < 7 h).

Age was analyzed as both a continuous and a categorical variable. A receiver operating characteristic (ROC) curve analysis was performed to identify the optimal cutoff value for predicting MetS. The analysis, conducted using MedCalc software (Version 23.1.3), identified 49 years as the optimal cutoff (area under the curve [AUC] = 0.581, sensitivity = 74.6%, and specificity = 39.1%). For interpretability and consistency with commonly used midlife classifications in epidemiological research, age was dichotomized as ≥ 50 years and < 50 years.

Physical activity was assessed according to the World Health Organization (WHO) guidelines, and individuals were classified as meeting or not meeting the minimum recommendation of 150 min of moderate‐intensity aerobic activity per week [9]. Sleep duration was categorized based on a 7‐h threshold, reflecting recommendations for adequate sleep among adults [16].

Vitamin D status was measured via serum 25‐hydroxyvitamin D [25(OH)D_3_] concentration and categorized as sufficient (≥ 20 ng/mL) or insufficient (< 20 ng/mL) based on commonly accepted clinical cutoffs [12]. BMI was categorized as < 25 or ≥ 25 kg/m^2^ according to the Asia‐Pacific obesity criteria [11]. History of physician‐diagnosed hypertension and diabetes was obtained through self‐report questionnaires administered during the health interview survey.

### 2.4. Outcome Variable

The primary outcome variable was the presence of MetS, defined based on the National Cholesterol Education Program Adult Treatment Panel III (NCEP ATP III) criteria, modified for the Asian population. Participants were classified as having MetS if they met three or more of the following criteria: abdominal obesity (waist circumference ≥ 90 cm for men and ≥ 85 cm for women), elevated triglycerides (≥ 150 mg/dL), reduced HDL cholesterol (< 40 mg/dL for men and < 50 mg/dL for women), elevated blood pressure (SBP ≥ 130 mmHg or DBP ≥ 85 mmHg or use of antihypertensive medication), and elevated fasting glucose (≥ 100 mg/dL or use of antidiabetic medication) [17].

### 2.5. Statistical Analysis

All analyses incorporated the survey’s complex design (sampling weights, strata, and primary sampling units). Participant characteristics by MetS status were summarized with Complex Samples Descriptives in IBM SPSS Statistics 30 (Windows; IBM Corp., Armonk, NY, USA). Continuous variables are presented as mean ± standard deviation (SD), and categorical variables are presented as unweighted frequencies and survey‐weighted percentages. Group differences used design‐based *t* tests for continuous variables and Rao–Scott chi‐square tests for categorical variables (Complex Samples Crosstabs). A survey‐weighted multivariable logistic regression (Complex Samples Logistic Regression) estimated adjusted odds ratios (ORs) with 95% confidence intervals (CIs), entering all prespecified covariates simultaneously; variances were computed by Taylor series linearization. Model performance metrics included Nagelkerke *R* ^2^ and the Hosmer–Lemeshow goodness‐of‐fit test adapted for complex samples. A two‐sided *α* = 0.05 denoted statistical significance. Domain (subpopulation) analysis was applied to restrict inference to adults aged 40–64 years while preserving survey weights.

### 2.6. Ethical Considerations

This secondary analysis used deidentified, publicly available data from the 2023 KNHANES provided by the KDCA. In KNHANES, participants provided written informed consent at the time of the original survey, and the research files released to investigators are deidentified to protect confidentiality. The KNHANES protocol is reviewed annually by the KDCA Institutional Review Board (IRB; e.g., IRB No. 2022‐11‐16‐R‐A). For this study, the IRB of Daejin University reviewed the protocol and granted an exemption (Approval No.: 1040656‐202511‐HR‐01‐09; Approval date: 2025‐06‐26). Because only deidentified public‐use data were analyzed, no additional consent from participants was required.

## 3. Results

### 3.1. Sociodemographic Characteristics

Applying survey weights and complex‐sample procedures, participants with MetS were older and more likely to be aged ≥ 50 years than those without MetS (both *p* < 0.001). Men were more prevalent in the MetS group than in the non‐MetS group (57.2% vs. 35.6%, *p* < 0.001). Participants with MetS were also less likely to have a college education (39.7% vs. 50.2%, *p* < 0.001), less likely to belong to the higher‐income group (65.0% vs. 70.0%, *p* = 0.014), and more likely to reside in rural areas (23.6% vs 17.2%, *p* < 0.001) (Table 1).

**TABLE 1:** Sociodemographic characteristics of participants by metabolic syndrome status (*N* = 2580).

Variables	No MetS (*n* = 1863)	MetS (*n* = 717)	*χ* ^2^/*t*	*p*
Age (mean ± SD)	52.25 ± 7.48	54.35 ± 6.93	6.54	< 0.001
Age ≥ 50 years, *n* (%)	1134 (60.9%)	535 (74.6%)	42.83	< 0.001
Sex (male), *n* (%)	664 (35.6%)	410 (57.2%)	98.87	< 0.001
≥ College education, *n* (%)	936 (50.2%)	285 (39.7%)	22.87	< 0.001
High income (top 50%), *n* (%)	1304 (70.0%)	466 (65.0%)	6.01	0.014
Rural residence, *n* (%)	321 (17.2%)	169 (23.6%)	13.53	< 0.001

*Note:* Values are survey‐weighted; *n* are unweighted counts. Group comparisons used design‐based *t*‐tests for continuous variables and Rao–Scott *χ* ^2^ tests for categorical variables (complex‐sample procedures). Systolic and diastolic blood pressure values represent the average of the second and third measurements. HDL cholesterol is presented separately for men and women because sex‐specific thresholds are used in lipid assessment. HDL, high‐density lipoprotein cholesterol; LDL, low‐density lipoprotein cholesterol; MetS, metabolic syndrome.

Abbreviations: BMI, body mass index; BP, blood pressure; SD, standard deviation.

### 3.2. Behavioral and Biological Characteristics

Using survey weights and complex‐sample procedures, current smoking was more prevalent among participants with MetS than those without MetS (25.5% vs. 15.1%, *p* < 0.001). In contrast, alcohol use did not differ between groups (*p* = 0.984). Participants with MetS were less likely to meet the WHO physical activity recommendations compared with those without MetS (27.6% vs. 36.8%, *p* < 0.001), whereas sleep duration did not show a significant difference (*p* = 0.172).

From a biological perspective, serum vitamin D levels were lower in participants with MetS than in those without MetS (23.09 ± 9.81 vs. 26.12 ± 42.95 ng/mL, *p* = 0.004), and the proportion of individuals with vitamin D sufficiency was also smaller in the MetS group (58.4% vs. 66.1%, *p* < 0.001) (Table 2).

**TABLE 2:** Comparison of behavioral and biological characteristics by the presence of metabolic syndrome (*N* = 2580).

Variables	No MetS (*n* = 1863)	MetS (*n* = 717)	*χ* ^2^/*t*	*p*
Current smoking, *n* (%)	281 (15.1%)	183 (25.5%)	38.26	< 0.001
Alcohol use (past year), *n* (%)	1422 (76.3%)	547 (76.3%)	0.00	0.984
Meets WHO PA, *n* (%)	686 (36.8%)	198 (27.6%)	19.49	< 0.001
Sleep duration ≥ 7 h, *n* (%)	1043 (56.0%)	380 (53.0%)	1.87	0.172
Serum vitamin D, ng/mL	26.12 ± 42.95	23.09 ± 9.81	2.87	0.004
Vitamin D ≥ 20 ng/mL, *n* (%)	1232 (66.1%)	419 (58.4%)	13.29	< 0.001

*Note:* Values are survey‐weighted; *n* are unweighted counts. Group comparisons used design‐based *t*‐tests for continuous variables and Rao–Scott *χ* ^2^ tests for categorical variables (complex‐sample procedures). Physical activity reflects meeting the World Health Organization recommendation. Vitamin D refers to serum 25‐hydroxyvitamin D [25(OH)D_3_]. MetS, metabolic syndrome. 25(OH)D_3_, 25‐hydroxyvitamin D.

Abbreviations: PA, physical activity; SD, standard deviation; WHO, World Health Organization.

### 3.3. Clinical Markers and Chronic Disease Status

Table 3 presents survey‐weighted clinical markers and chronic disease indicators according to MetS status. Participants with MetS had substantially higher adiposity, with BMI significantly greater than that of those without MetS (27.08 ± 3.35 vs. 23.04 ± 3.10 kg/m^2^, *p* < 0.001).

**TABLE 3:** Clinical and chronic disease characteristics by metabolic syndrome (MetS) status (*N* = 2580).

Variables	No MetS (*n* = 1863)	MetS (*n* = 717)	*χ* ^2^/*t*	*p*
BMI (kg/m^2^)	23.04 ± 3.10	27.08 ± 3.35	−27.86	< 0.001
Systolic BP (mmHg)	114.75 ± 13.63	125.58 ± 13.75	−17.94	< 0.001
Diastolic BP (mmHg)	74.22 ± 9.33	79.66 ± 8.63	−13.99	< 0.001
Fasting glucose (mg/dL)	96.38 ± 16.91	114.74 ± 30.09	−15.43	< 0.001
Triglycerides (mg/dL)	104.56 ± 55.27	204.02 ± 139.99	−18.48	< 0.001
LDL (mg/dL)	107.80 ± 39.96	120.55 ± 35.60	5.48	< 0.001
HDL (mg/dL), men	55.02 ± 13.59	43.71 ± 10.51	14.40	< 0.001
HDL (mg/dL), women	65.99 ± 14.49	49.96 ± 12.59	17.75	< 0.001
Hypertension, *n* (%)	160 (8.6%)	264 (36.8%)	235.18	< 0.001
Diabetes, *n* (%)	99 (5.3%)	161 (22.5%)	167.86	< 0.001

*Note:* Values are survey‐weighted; *n* are unweighted counts. Group comparisons used design‐based *t*‐tests for continuous variables and Rao–Scott *χ* ^2^ tests for categorical variables (complex‐sample procedures). Systolic and diastolic blood pressure values represent the average of the second and third measurements. HDL cholesterol is presented separately for men and women due to sex‐specific diagnostic criteria for metabolic syndrome. HDL, high‐density lipoprotein cholesterol; MetS, metabolic syndrome.

Abbreviations: BMI, body mass index; BP, blood pressure; SD, standard deviation.

Glycemic and lipid profiles were also less favorable in the MetS group, with higher fasting glucose (114.74 ± 30.09 vs. 96.38 ± 16.91 mg/dL, *p* < 0.001) and triglyceride levels (204.02 ± 139.99 vs. 104.56 ± 55.27 mg/dL, *p* < 0.001). Blood pressure was consistently elevated among participants with MetS, with higher systolic (125.58 ± 13.75 vs. 114.75 ± 13.63 mmHg, *p* < 0.001) and diastolic values (79.66 ± 8.63 vs. 74.22 ± 9.33 mmHg, *p* < 0.001).

Chronic conditions were also more prevalent in the MetS group, with higher rates of diabetes (22.5% vs. 5.3%, *p* < 0.001) and hypertension (36.8% vs. 8.6%, *p* < 0.001). In addition, HDL cholesterol levels were lower among participants with MetS in both men and women, consistent with the characteristic dyslipidemia associated with MetS (Table 3).

### 3.4. Multivariable Logistic Regression Identifying Predictors of MetS

Table 4 presents the survey‐weighted multivariable logistic regression identifying independent predictors of MetS, with all prespecified covariates entered simultaneously and variances estimated using Taylor series linearization. The model demonstrated acceptable explanatory power (Nagelkerke *R* ^2^ = 0.342) and good fit (Hosmer–Lemeshow *χ* ^2^ = 16.104, df = 8, and *p* = 0.081).

**TABLE 4:** Multivariable logistic regression analysis of factors associated with metabolic syndrome.

Variables	B	*p*	Exp (B)	95% CI for exp (B)
Vitamin D ≥ 20 ng/mL	−0.240	0.024	0.787	0.639–0.969
Age ≥ 50 years	0.702	< 0.001	2.018	1.596–2.552
Male sex	−0.687	< 0.001	0.503	0.399–0.633
≥ College education	−0.228	0.044	0.796	0.638–0.993
High income	−0.129	0.258	0.879	0.702–1.099
Rural residence	0.301	0.018	1.351	1.053–1.733
Alcohol use	−0.129	0.304	0.879	0.687–1.124
Current smoking	0.394	0.005	1.483	1.128–1.951
Meets WHO PA	−0.364	0.001	0.695	0.557–0.867
Sleep ≥ 7 h/day	−0.021	0.835	0.979	0.800–1.198
BMI ≥ 25 kg/m^2^	2.182	< 0.001	8.867	7.210–10.904
Constant	−1.300	< 0.001	0.273	—

*Note:* Odds ratios and 95% confidence intervals are from Complex Samples Logistic Regression with Taylor series linearization; all prespecified covariates were entered simultaneously. Model performance: Nagelkerke *R* ^2^ = .342; design‐based goodness‐of‐fit (Hosmer–Lemeshow *χ* ^2^ = 16.104, df = 8, *p* = 0.081). MetS, metabolic syndrome.

Abbreviations: BMI, body mass index; CI, confidence interval; OR, odds ratio; PA, physical activity; WHO, World Health Organization.

Older age (≥ 50 vs. < 50 years) was associated with higher odds of MetS (OR = 2.02, 95% CI 1.60–2.55, and *p* < 0.001), whereas male sex was associated with lower adjusted odds (OR = 0.50, 95% CI 0.40–0.63, and *p* < 0.001). Rural residence (OR = 1.35, 95% CI 1.05–1.73, and *p* = 0.018) and current smoking (OR = 1.48, 95% CI 1.13–1.95, and *p* = 0.005) were positively associated with MetS, while meeting the WHO physical activity recommendation was protective (OR = 0.70, 95% CI 0.56–0.87, and *p* = 0.001).

Higher educational attainment (college or above) was associated with lower odds of MetS (OR = 0.80, 95% CI 0.64–0.99, and *p* = 0.044). BMI ≥ 25 kg/m^2^ emerged as the strongest predictor (OR = 8.87, 95% CI 7.21–10.90, and *p* < 0.001), and vitamin D sufficiency (≥ 20 ng/mL) was also associated with reduced odds (OR = 0.79, 95% CI 0.64–0.97, and *p* = 0.024). Income, alcohol use, and sleep duration were not significantly associated with MetS (Table 4).

## 4. Discussion

### 4.1. Overview of Key Findings

This study aimed to estimate the prevalence of MetS and identify its independent determinants among adults aged 40–64 years using nationally representative data from South Korea. In this analysis, the prevalence of MetS was 27.8%, and several independent determinants emerged with clear relevance for nursing practice. Higher odds of MetS were observed with older age within the target range, rural residence, current smoking, not meeting the physical activity recommendation, vitamin D insufficiency, and BMI ≥ 25 kg/m^2^, whereas higher educational attainment was protective. BMI was the strongest predictor. Taken together, these findings delineate a focused set of modifiable risks that can be addressed through nurse‐led screening, brief counseling, and referral in primary and community care.

### 4.2. Sociodemographic Determinants of MetS

Age‐related increases in MetS are biologically plausible, reflecting cumulative sarcopenia, visceral adiposity, and chronic low‐grade inflammation that reduce insulin sensitivity and worsen lipid and blood pressure profiles over time [18]. Sex differences in midlife often favor a higher burden of MetS among men; however, this difference may narrow or reverse after menopause, when hormonal changes in women may contribute to increased visceral adiposity and worsening metabolic risk profiles [5]. The inverse association with education aligns with literature linking educational attainment to health literacy, preventive service use, and healthier environments, suggesting that social resources shape cardiometabolic trajectories before disease is clinically apparent [6]. Rural residence independently predicted MetS in our model; documented barriers—longer travel distances, fewer built‐environment supports for activity, and limited prevention services—likely contribute, underscoring the need for equity‐oriented implementation strategies [6, 19].

### 4.3. Behavioral Determinants

Behavioral factors offer immediate leverage for intervention. Current smoking was associated with higher odds of MetS, consistent with mechanistic work showing that tobacco exposure promotes oxidative stress, endothelial dysfunction, dyslipidemia, and preferential central fat accumulation—pathways that aggravate metabolic clustering [20]. Conversely, meeting the WHO physical activity recommendation is linked to improved insulin sensitivity, blood pressure, and lipid profiles, and to reductions in visceral adiposity; these physiologic effects plausibly translate into lower MetS risk [9, 21]. Alcohol use and sleep duration were not independently associated after adjustment, which may reflect measurement limitations rather than null effects. Dose and pattern matter for alcohol, and sleep health encompasses duration, regularity, and quality; future studies should incorporate more granular measures to clarify these relationships in midlife.

### 4.4. Biological Determinants

Biological indicators reinforce established pathophysiology. BMI ≥ 25 kg/m^2^ dominated the risk profile, consistent with evidence that excess adiposity—particularly visceral fat—drives insulin resistance, ectopic lipid deposition, and proinflammatory signaling [22]. Vitamin D sufficiency was associated with lower odds of MetS. Although causality remains debated, proposed mechanisms include effects on pancreatic β‐cell function, immune modulation, and systemic inflammation; several observational syntheses report inverse associations between 25‐hydroxyvitamin D and MetS or its components [23]. Given the high frequency of low vitamin D status reported in adults in this region, attention to vitamin D within routine assessments may be warranted, while recognizing that randomized trial evidence for supplementation on MetS endpoints is still evolving [24]. The clustering of diabetes and hypertension with MetS in our data is expected and supports integrated cardiometabolic risk management across primary and community settings [25].

### 4.5. Implications for Nursing Practice

These findings translate into practical actions. Routine midlife encounters can support rapid risk stratification—waist circumference and blood pressure measurement, documentation of smoking and physical activity status against the WHO threshold, and consideration of vitamin D testing where resources allow [21]. Brief counseling can be delivered efficiently with behavior‐change techniques (goal setting, action planning, and follow‐up) and referral to cessation services, community exercise programs (e.g., walking groups and step‐count prescriptions), nutrition support, and weight‐management resources [26]. The observed social patterning argues for equity‐oriented delivery: outreach clinics, mobile services, and partnerships with local organizations can help overcome access barriers in rural communities and among adults with lower educational attainment. Embedding midlife MetS assessment into routine quality indicators and electronic prompts may standardize practice at scale [27].

### 4.6. Strengths and Limitations

Strengths of this study include contemporary, nationally representative data; standardized clinical measures; and survey‐weighted analyses that respected the complex sampling design, yielding population‐relevant estimates. Limitations include the cross‐sectional design, which precludes causal inference; reliance on some self‐reported variables with potential misclassification; and dichotomization of sleep and vitamin D, which may obscure nonlinear associations. Diet and sleep quality were not captured with sufficient granularity, and residual confounding is possible despite adjustment. In addition, detailed geographic information was not available in the dataset, limiting the ability to examine regional variation in greater depth. Although urban–rural residence was included as a proxy for geographic differences, more granular geospatial measures were not available.

### 4.7. Future Directions

Future work should test nurse‐led intervention bundles that integrate smoking cessation, activity promotion, weight management, and, where appropriate, vitamin D optimization; incorporate richer measures of alcohol patterning and sleep health; and evaluate implementation outcomes (reach, adoption, fidelity, and cost‐effectiveness), with stratification by residence and educational attainment and meaningful community engagement.

## 5. Conclusion

In this nationally representative sample of adults aged 40–64 years, MetS was strongly associated with modifiable factors, including smoking, insufficient physical activity, and excess adiposity, as well as sociodemographic characteristics such as education and rural residence. BMI emerged as the strongest predictor, while higher educational attainment and sufficient vitamin D levels were associated with lower odds of MetS. These findings highlight key targets for prevention and support the role of nurse‐led interventions in reducing cardiometabolic risk in midlife populations.

**Nomenclature**
AUC: Area under the curve
BMI: Body mass index
CI: Confidence interval
HDL: High‐density lipoprotein
KNHANES: Korea National Health and Nutrition Examination Survey
LDL: Low‐density lipoprotein
MetS: Metabolic syndrome
OR: Odds ratio
PA: Physical activity
SD: Standard deviation
WHO: World Health Organization

## Author Contributions

Conceptualization: Kyeongmin Jang.

Methodology: Kyeongmin Jang.

Formal analysis: Kyeongmin Jang.

Investigation: Kyeongmin Jang.

Data curation: Kyeongmin Jang.

Writing–original draft: Kyeongmin Jang.

Writing–review and editing: Kyeongmin Jang.

Visualization: Kyeongmin Jang.

Supervision: Kyeongmin Jang.

## Funding

No external funding was received for this research.

## Ethics Statement

This study used anonymized, publicly available data from the 2023 Korea National Health and Nutrition Examination Survey (KNHANES), conducted by the Korea Disease Control and Prevention Agency (KDCA). The KNHANES protocol is reviewed annually by the KDCA Institutional Review Board (IRB No. 2022‐11‐16‐R‐A). In accordance with the institutional policy for secondary analyses of deidentified public‐use data, the Daejin University IRB granted exemption (Approval No. 1040656‐202511‐HR‐01‐09). No additional consent from participants was required.

## Consent

Please see the Ethics Statement.

## Conflicts of Interest

The author declares no conflicts of interest.

## Data Availability

The KNHANES public‐use data analyzed in this study are deidentified and openly available from the Korea Disease Control and Prevention Agency through the official KNHANES website.
